# Automated versus human scoring of the Rey-Osterrieth Complex Figure Test: a rapid review

**DOI:** 10.3389/fpsyt.2025.1746720

**Published:** 2026-01-14

**Authors:** Sander Lindholm Andersen, Astri J. Lundervold, Eivind Haga Ronold

**Affiliations:** 1 Division of Psychiatry, Haukeland University Hospital, Bergen, Norway; 2 Department of Biological and Medical Psychology, Faculty of Psychology, University of Bergen, Bergen, Norway; 3 Department of Neurology, Haukeland University Hospital, Bergen, Norway; 4 Department of Radiology, Mohn Medical Imaging and Visualization Centre, Bergen, Norway

**Keywords:** artificial intelligence, automatic scoring, deep learning, diagnostic tool, neuropsychological assessment, Rey Osterrieth Complex Figure Test, validation

## Abstract

**Introduction:**

Despite digital advances in healthcare, clinical neuropsychology has been slow to adopt automated assessment tools. Automated scoring of the Rey-Osterrieth Complex Figure Test (ROCFT) could enhance efficiency and consistency in evaluating quantitative and qualitative aspects of the figure. However, clinical utility and accuracy compared to traditional scoring methods remain unclear.

**Objective:**

To evaluate whether digital automated scoring systems provide accuracy, reliability, and clinical utility equal to or superior to traditional clinician-driven scoring of the ROCFT.

**Methods:**

A rapid review following the PRISMA guidelines was conducted. PubMed and Web of Science were searched from January 1, 2015, to October 12, 2025, and included recent studies that benchmark automated scoring against human raters.

**Results:**

The review identified five articles, three with deep-learning approaches and two that used rule-based algorithms. Together, they analysed more than 41,000 ROCFT drawings with diverse capture methods. Overall, well-designed automated systems can achieve, and in some cases surpass, expert-level performance. The algorithmic approaches demonstrated close agreement with trained raters and reproducible outputs, with discrepancies primarily emerging in atypical drawings. Deep-learning models achieved high concordance with expert scoring when image quality was adequate, and training data were well-labelled. However, performance varied with data quality and distribution of scores.

**Discussion and conclusion:**

This review demonstrates that digital automated ROCFT scoring achieves accuracy and reliability compared to traditional clinician ratings, with well-designed systems occasionally surpassing human performance. Expected advances in artificial intelligence and automation could further enhance clinical neuropsychology into the 21st century. However, clinical implementation faces several constraints: heterogeneous training datasets, limited evidence of usefulness across disorders, and lack of independent validation. Automated scoring should thus augment, not replace, clinical judgement. To address these limitations, future research should strive to establish disorder−specific norms, conduct independent validation in real-world clinical settings, and develop human-in-the-loop pipelines that combine automated efficiency with clinical oversight. Responsible implementation will require explicit governance frameworks that regulate data use and sharing, address privacy and ‘mental data’ concerns. These advances would strengthen the evidence-base and utility for automated ROCFT scoring and support its responsible integration into neuropsychological practice.

## 1 Introduction

While clinical neuropsychology has long been the gold standard for measuring cognition in clinical practice, its methods have evolved more slowly than the digital transformation in other areas of health care (1, 2). Tests are still mostly administered on paper, scored by hand, at least partly dependent on the rater’s training and time. The Rey-Osterrieth Complex Figure Test (ROCFT) exemplifies both the strengths and constraints of this approach (3). It is a well-established test for assessing visuoconstruction, executive planning, and visual memory by requiring participants to copy and recall a complex figure, where 18 parts are given 0–2 points, with 36 points in total (4). However, neuropsychological test administration and scoring are labor-intensive, and inter-rater reliability remains problematic (5). The value of neuropsychological tests depends on norms for specific conditions. Tests require demographically stratified, disorder-relevant norms to yield valid inferences across age, education, and cultural groups (6). To address these challenges, proponents of a Neuropsychology 3.0 framework argue that the way forward is to transform test results into data streams that can be audited, compared across sites, and integrated with electronic medical records and decision support (7). Thus, automated ROCFT scoring can save time and enhance measurement fidelity: item-level outputs and process-features can be mapped to explicit cognitive constructs, improving transparency, inter-rater consistency, and longitudinal comparability and clinical utility while preserving clinician oversight. Demand for assessments is rising as populations age, and as neurological and psychiatric conditions are recognized and treated earlier (8). Thus, improving assessment and scoring efficiency becomes increasingly important for the field. Within this landscape, automated scoring of tests such as the ROCFT may offer faster feedback to the patients, more standardized and sharable documentation in clinical teams, and structured data supporting ongoing research and service improvement.

Early work laid the ground for automated ROCFT scoring. Canham et al. (9) proposed a rule-based computer-vision pipeline that identified basic geometric shapes in scanned drawings and matching it to the Osterrieth scoring system. Although promising, drawings with missing or highly distorted pieces still needed a human rater. Given recent technological advances, previous shortcomings of digital scoring can be traversed. Automated scoring, particularly approaches that leverage artificial intelligence (AI) and deep learning (DL), offers a novel path to optimize workload while preserving the test’s clinical utility. Automation can deliver instant scoring, reducing turnaround time and freeing clinician time for interpretation of results and patient feedback (10). At a deeper level, digital capture of patient drawings could enable systematic extraction of item-level errors and process features such as stroke order, pauses, spatial organization, and timing, which opens possibilities of quantification and novel ways of interpretation and clinical utility (11). Standardization is another advantage. Automated pipelines apply the same rules across drawings, clinics, and time, which can improve inter-rater consistency and reliability (5) and quality assurance (12). Additional benefits concern scalability and access. Archives of patient records containing ROCFT drawings, are rarely re-analysed. Image-based automation can unlock these archives for retrospective research and service evaluation, while tablet-based capture can streamline prospective clinical workflows. In settings with limited neuropsychology staffing, automated scoring may support triage, such as flagging concerning results (13). Automated scoring systems can serve as a safety net for practitioners by providing an independent, objective score that serves as verification, particularly for borderline or complex cases. Also, systems trained on large datasets can flag atypical drawings that deviate from typical patterns, and discrepancies between automated and manual scores provide feedback that can refine clinical judgment over time. While empirical evidence for these specific applications in ROCFT is still emerging, these mechanisms parallel established clinical decision support systems in other medical contexts (14).

When embedded in human-in-the-loop workflows, automation becomes a second opinion that accelerates routine cases while supporting evaluation of atypical cases for closer review. However, successfully implementing such systems requires evidence about their performance relative to human raters and careful consideration of practical constraints. The present rapid review addresses this need by examining the following question: Do digital automated scoring systems provide accuracy, reliability, and clinical utility superior to traditional clinician-driven scoring of the ROCFT? To answer this question, we synthesize recent comparisons between automated and human scoring, and evaluate these findings regarding workflow, generalizability, and governance. The study aims to clarify whether automated ROCFT scoring is ready to augment clinical practice and to identify what evidence and safeguards are needed to support responsible integration.

## 2 Methods

A review following Preferred Reporting Items for Systematic Reviews and Meta-Analyses guidelines (15), adapted for rapid reviews, was conducted. We pre-specified five inclusion criteria: (1) published in English, (2) including a form of digitally automated ROCFT scoring system (or variation), (3) reporting a head-to-head comparison between a human/expert rater and an automated system, (4) peer-reviewed research paper published in an international journal, and (5) published between 2015 and 2025. Publication date was restricted because relevant applications of modern computerized scoring have largely emerged within the past decade. Earlier work, such as Canham et al. (9), mainly comprised proof-of-concept systems developed under substantially different hardware and software constraints. This better reflects contemporary technological possibilities. Papers included digital variants and automated scorers using the Quantitative Scoring System (QSS; 0–36) totals or equivalent element-level scoring and studies evaluating automated clinical decisions derived from ROCFT drawings.

**Figure 1:**
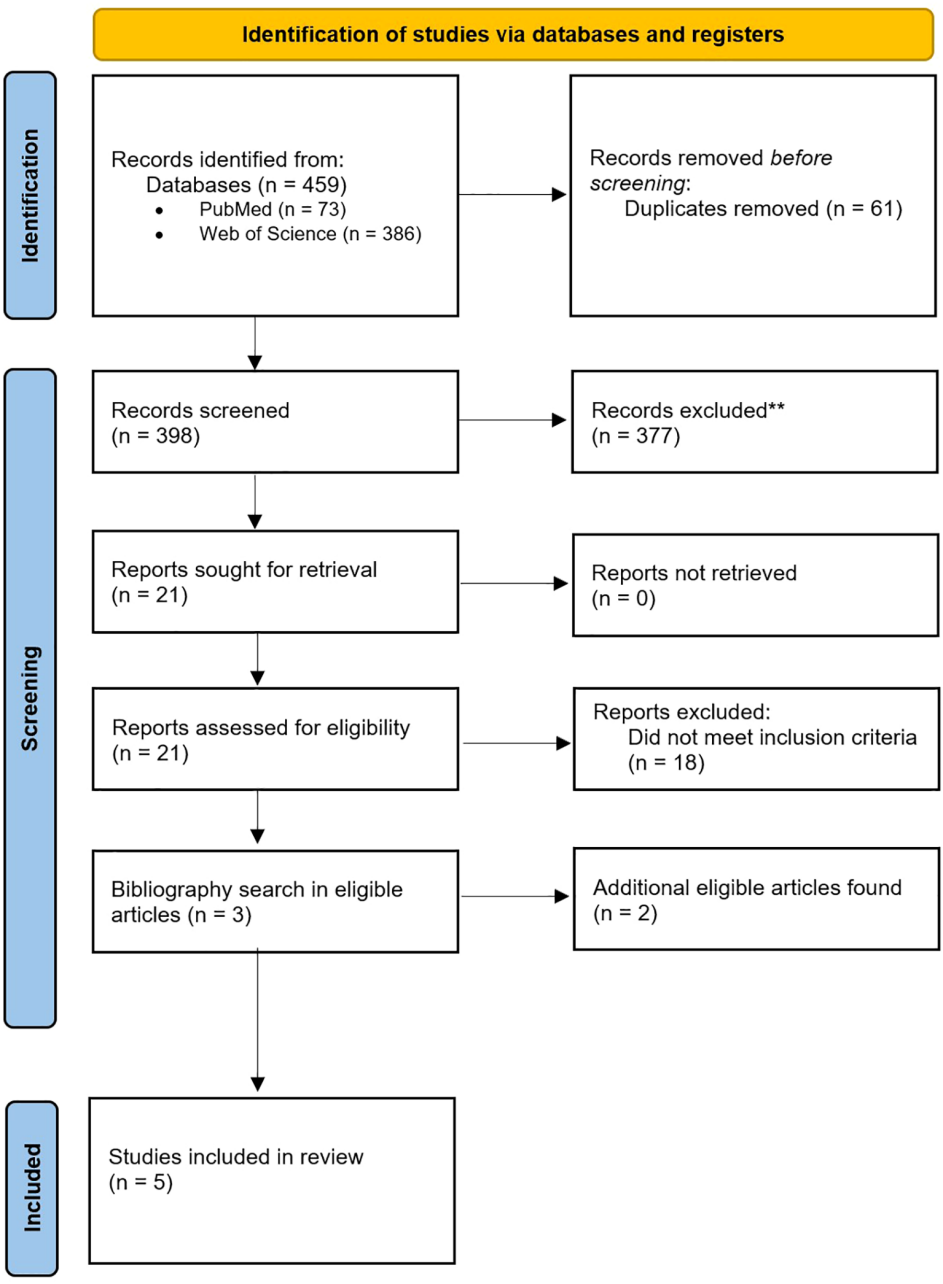
PRISMA 2020 flow diagram showing the identification, screening, eligibility assessment, and inclusion of studies in this rapid review (PubMed and Web of Science; final included studies, n = 5).

PubMed and Web of Science were searched, from January 1, 2015, to October 12, 2025. A total of 459 papers were identified, and 61 duplicates were removed. The first author screened titles/abstracts and excluded 377 records according to the review criteria. Among the reports assessed for eligibility, 18 did not meet inclusion criteria. Finally, the bibliographies of all eligible articles were systematically searched to identify further eligible work. See **Figure 1** for PRISMA flow chart.

## 3 Results

Five studies were identified including data on >41,000 ROCFT drawings. Details about the five studies are presented in **Table 1**.

**Table 1:** Characteristics and key findings of included studies comparing automated Rey–Osterrieth Complex Figure Test (ROCFT) scoring with human raters, summarizing study design, model type (deep learning vs algorithmic/classical computer vision), sample size, comparison approach, and main performance outcomes (e.g., MAE/MSE, ICC/PCC, ROC/AUC).

Authors (year)	Design	Method	Human group	Automation model	Deep Learning	n (people/drawings)	Comparison	Results
Langer et al. (12)	Large retrospective dataset + preregistered prospective validation	Multi-head CNN (item-wise classification + total-score regression), robustness analyses	Clinicians from six clinics; crowdsourced online raters	CNN system scoring 18 elements + total QSS from images	Yes (DL)	>20,000 drawings; ~4,030 with clinician scores; ~2,498 prospective	AI total score vs expert mean; agreement vs inter-expert variability	Model outperformed clinicians (MAE ≈ 1.11–1.16 vs ≈ 2.15) and online raters (≈ 2.41); performance replicated prospectively
Guerrero-Martín et al. (16)	Benchmark dataset + human–machine comparison	Multiple CNNs (Sketch-a-Net, EfficientNet, MobileNetV2, Inception); 16-fold CV	Two independent experts scored a subset (n = 185)	CNNs trained on ROCFD528 copies to predict QSS	Yes (DL)	528 drawings total; 185 expert-scored subset	Model MAE/MSE vs clinicians and vs online raters (item-wise and total)	Humans stronger at low scores; best DL approaches/surpasses experts at higher scores; overall best model MAE ≈ 3.45 on a hard, imbalanced dataset
Park et al. (13)	Retrospective model development + external expert test	DenseNet CNN regressor on scanned RCFT copy/immediate/delayed images with expert QSS labels	Five experienced psychologists independently scored 150 external images	CNN (DenseNet) predicting 36-point QSS from images	Yes (DL)	6,680 people/20,040 drawings; 150 drawings (external expert test)	Models vs experts (MAE, RMSE, R², PCC) incl. per-score analyses	External test: MAE ≈ 0.64; R² ≈ 0.994. Authors conclude no fundamental difference between expert and AI ratings; suitable for large-scale use
Webb et al. (11)	Validation study in healthy adults and stroke	Tablet-based OCS-Plus Complex Figure; automated program vs manual	Two trained psychologists (manual copy/recall scoring)	Rule-based element extraction and scoring	No (algorithmic)	Healthy n = 261; Stroke n = 203 (total = 464)	Automated vs manual scores (ICC) and impairment classification (ROC/AUC)	ICC ≈.83 vs manual; element detection sens/spec ≈ 92%/90%; diagnostic sens 80%/spec 93.4% (AUC 86.7%) vs manual categories
Sangiovanni et al. (17)	Pilot HRI validation (robot administering tests)	Classical CV (OpenCV) on camera-captured ROCF drawings	One psychologist scored all drawings	Region-matching metric with penalization factor and global metric	No (classical CV)	n = 37 participants; copy & recall captured by robot	Automatic ROCF scores vs psychologist’s scores (r, ρ, α; slope tests)	Strong correlations (e.g., r up to.79; ρ up to.84; α up to.87*. Global score best calibrated

Three studies used automated scoring based on DL (12, 13, 16), while two used algorithmic approaches combined with digital tablets and styluses or digitized images of the drawings (11, 17). Studies differed in drawing capture methods (scanner images, photographs, or tablet input); predictors (QSS total scores or item-level scores); human benchmarks (expert raters, practicing clinicians, or crowd raters); and participant types (healthy adults, stroke survivors, patients with neurological/psychiatric diagnoses). Across the five studies, capture modalities included scanned paper drawings (12, 13, 16), camera-based images (17), and digital tablet input (11). Predictors also varied: Webb et al. (11) and Park et al. (13) focused on automated estimation of totals comparable to QSS. Langer et al. (12) and Guerrero-Martín et al. (16) trained models on element-level labels and then aggregated these to total scores. Human benchmarks ranged from a single experienced clinician (17) to panels of expert raters (11–13, 16) and crowdsourced raters (12). Samples likewise differed, including healthy adults, stroke survivors, and individuals across the cognitive spectrum from normal cognition through mild cognitive impairment to dementia (11–13, 16, 17).

Two studies show that algorithmic automation align well with human scoring. Webb et al. (11) validated a tablet-based scoring system for the Oxford Cognitive Screen-Plus complex figure task against human scoring. Their algorithm segmented the digital drawing into the 18 predefined elements, applied geometric and positional rules to classify each element as present, distorted, or misplaced, and then aggregated these decisions to copy and recall totals. Automated scores correlated closely with manual ratings, with intraclass correlations around .83 and element-detection sensitivity and specificity of roughly 92% and 90%. Discrepancies occurred mainly in atypical drawings.

Sangiovanni et al. (17) programmed a human-like robot to administer a cognitive test battery, including ROCFT. Drawings captured on camera were scored using two complementary automated metrics. The element-matching score quantified how well predefined ROCFT elements in the drawing overlapped with a reference template, providing an approximation of item-level accuracy. In parallel, a global similarity score evaluated the overall correspondence between the full drawing and the template, thereby capturing large-scale distortions, misconfigurations, or omissions that may not be fully reflected by element-level overlap alone. Automated scores showed strong agreement with the expert ratings, with Pearson correlations up to r .79, Spearman ρ up to .84, and internal consistency estimates (Cronbach’s α) up to .87. The global score provided the best calibration against the human benchmark. Overall, these results demonstrate that transparent, rule-based algorithmic scoring can achieve clinically acceptable agreement with human scoring.

In all included DL studies, automated scoring achieved high concordance with expert scoring when image quality was adequate, and training labels were accurate. In some cases, automated ROCFT scoring matched or exceeded human performance. Park et al. (13) trained a convolutional neural network (CNN) on 20,040 drawings. The model was then tested on an external set of 150 drawings scored by five psychologists (defined as gold standard). The model’s predicted QSS totals showed very small mean absolute error (MAE ≈ 0.64), high explained variance (R² ≈ 0.99), and Pearson correlations comparable to inter-expert agreement. Accuracy of the DL scores was comparable to that of human experts. Langer et al. (12) trained a multiheaded CNN on >20,000 drawings (≈ 4,000 with clinician scores) and prospectively validated it on ≈ 2,500 drawings. In both retrospective and preregistered prospective evaluations, the model outperformed clinicians and crowdsourced raters on absolute error (MAE ≈ 1.11–1.16 vs ≈ 2.15 for clinicians and ≈ 2.41 for crowdsourced raters). Guerrero-Martín et al. (16) used the open dataset ROCFD528 to train their models (528 drawings). When comparing multiple CNNs against two expert raters, expert raters showed superior performance on lower score ranges, whereas the best model approached or exceeded expert performance at higher scores, with a best-case MAE around 3.45 on a deliberately challenging, imbalanced dataset. Collectively, these studies suggest that CNN-based scoring can achieve expert-level accuracy under optimal conditions, though performance is sensitive to dataset diversity.

## 4 Discussion

This rapid review addressed whether digital automated ROCFT scoring provides accuracy, reliability, and clinical utility superior to traditional clinician-driven scoring. The evidence shows that automated systems achieve comparable performance to human raters, with some studies demonstrating superior accuracy under optimal conditions. While not categorically superior in all contexts, well-designed digital systems match human-level performance while offering distinct advantages in standardization, speed, and auditability.

### 4.1 Current state of automated scoring

While the results presented in the included articles are encouraging, we identified only five articles that directly compared automated scoring to human ratings (11–13, 16, 17). Although automated scoring systems could provide multiple clinical benefits, including faster and more consistent scoring, improved capacity for large-scale analysis of both new and archived data, and standardized workflows across clinical sites, most systems have not been tested using external data by independent research teams.

Beyond the reviewed studies, other work has used digital ROCFT variants to extract process-level features without focusing on automated QSS scoring, also introducing novel techniques and applications. Prange and Sonntag (18) recorded ROCFT with a digital stylus to extract temporal and kinematic features from each stroke, enabling automatic classification of cognitive status without manual scoring. Similarly, Savickaite et al. (19) demonstrated how a tablet-based ROCFT can yield performance metrics (e.g. accuracy, organizational strategy, and motor patterns) and revealed group differences, that traditional methods might overlook. In addition, Kim et al. (20) found that while standard ROCFT scores did not distinguish between certain patient subgroups, gaze metrics did show divergent cognitive approaches (e.g. frequent fixations and switching in early-onset Alzheimer’s disease). Thus, combining technologies might augment clinical practice.

Importantly, DL has enabled diagnostic applications that extend substantially beyond automated scoring. While rule-based and classical ML approaches focus on replicating clinician scoring behavior, DL approaches can discover complex, non-linear patterns in ROCFT drawings that may not be captured by conventional scoring systems. Large-scale ROCFT datasets have been used to train deep neural networks that assist in diagnosing dementia (21) and identifying at-risk individuals (22, 23). These DL models leverage CNN-extracted features, capturing subtle drawing characteristics such as spatial relationships, element sequencing, and execution dynamics, that exceed what human raters typically quantify. By using these hierarchical feature representations or element-level outputs as inputs to diagnostic classifiers, DL transforms the ROCFT from a psychometric instrument into a multidimensional digital biomarker, extending applications beyond psychometric assessment into clinical diagnostic and prognostic domains.

### 4.2 Advantages of automated scoring

Automated scoring offers several advantages over manual ROCFT scoring, particularly speed and consistency. Manual scoring is labor-intensive and dependent on trained experts, which constrains throughput in hospital settings (24), whereas automated systems can alleviate this pressure (12). Decision-support pipelines can label each of the 18 ROCFT elements as omitted, distorted, misplaced, or correct, preserving error types that are lost in the traditional 0–36 total and providing model explanations that clarify which features drive diagnostic predictions (25). Tablet-based approaches extend this further by extracting time-stamped, spatial, procedural, and kinematic indices that correlate with conventional tests targeting the same constructs (26, 27) and build on prior work using digital drawing to assess fine motor skills and handwriting in conditions such as Parkinson’s disease (28). For diagnostic use, automated systems can combine element-level judgements and process metrics (e.g., stroke order, pauses, spatial organization) into structured, auditable outputs that feed classifiers, support group comparisons, and align with standard scoring for clinical deployment (11–13, 25).

### 4.3 Challenges and controversies

Applying DL to clinical data faces concrete barriers that indicate why deployment remains limited. Datasets are often too small or noisy for robust training, and privacy regulations such as GDPR, especially for health data, constrain cross-border sharing and cloud processing without strong safeguards. ROCFT drawings can constitute biometric/behavioral data, attracting additional restrictions analogous to handwriting-based identification (29). Emerging concepts like ‘mental privacy’ suggest that AI applied to neurodata may require protections beyond current AI frameworks (30), making data pooling, external validation, and iterative model updates more complex and slowing translation into routine care.

While current results demonstrate technical feasibility, acceptance of automated scoring by clinicians and its integration into clinical workflows remain a potential challenge. Langer et al. (12) mention that their application is in beta testing, indicating ongoing efforts to address this issue. However, currently none of the papers in our review address pathways for regulatory approval. Developers would need formal clinical evaluation demonstrating safety, performance, generalizability, and documentation on data protection, bias mitigation, human-in-the-loop use, and post-marked monitoring. Until such requirements are met, deployment will likely be limited to research rather than clinical practice.

Training AI requires large datasets, and a model constructed from the ground-up requires larger datasets until reaching a plateau (31). Pre-trained models can be fine-tuned with smaller datasets. In vision tasks, Shahinfar et al. (32) showed a robust logarithmic relationship between per-class sample size and accuracy/recall/precision, with diminishing returns beyond approximately 150–500 labelled images per-class in balanced designs. For automatic scoring of the ROCFT, this suggests prioritizing high-quality, diverse exemplars per scoring category, targeting a few hundred labelled drawings where feasible, and investing in curation and external validation. Dataset diversity is also important for transportability, as they are generally not accessible for most hospitals (33), raising questions about the feasibility of implementing AI scoring systems in smaller clinical settings with limited data access. A potential solution would be for hospitals to rely on data sharing.

Beyond data volume, process interpretability poses another challenge. Traditional machine learning methods require handcrafted features, which can provide some degree of interpretability for clinicians when the features themselves are clinically meaningful. DL learns from raw pixel data, achieving higher accuracy, but often functions as a "black box" with limited explainability. Clinical choice depends on context: DL suits high-volume screening where accuracy is paramount; more transparent approaches suit forensic, teaching, or resource-limited settings requiring clear decision pathways. Hybrid approaches combining DL feature extraction with interpretable scoring may balance accuracy and explainability (34). Both automated scoring and diagnostic classification can use either approach but serve distinct purposes: measurement versus clinical decision support.

Generalizability is important and training and evaluation should therefore demonstrate stable performance across age, cognitive status, and cultural backgrounds. Concrete strategies include assembling large, heterogeneous datasets with prospective and external testing (12), and reporting subgroup performance across copy/recall conditions and clinical strata (13). Transfer can be further improved by multi-stage fine-tuning, e.g., by pre-training on large datasets before ROCFT specialization (33). Finally, element-level outputs and model explanations make decisions traceable and biases visible, allowing clinicians to verify why a case was flagged (25).

This relates to risk for bias, such as under-representation of key subgroups. Additional threats include label bias (inconsistent rater rules) and domain shift (deployment images different from training), both of which could reduce accuracy. Mitigations include using multiple independent raters, stress-testing shifts and training with varied captures, routing low-confidence or out-of-distribution cases to a human-in-the-loop, and pre-specifying subgroup metrics (age, education, diagnosis, handedness/motor impairment) in advance of analysis. Together, these practices align automated scoring with clinical standards while preserving clinician oversight (12, 13, 25, 33) and thus should be implemented for clinical augmentation.

Comprehensive sensitivity for automated detection of neurological and psychiatric disorders remains limited. Langer et al. (12) reported such sampling, but did not break down diagnoses in detail, underscoring the need for disorder-specific evidence. Nevertheless, some recent studies have expanded diagnostic coverage: Di Febbo et al. (25) and Park et al. (13) both included participants across cognitive status categories (healthy, MCI, and dementia). Webb et al. (11) compared healthy adults with stroke survivors, and Hyun et al. (35) contrasted adolescents with ADHD to matched controls using a digital ROCFT variant. Frigeni et al. (27), Guerrero-Martín et al. (16), Petilli et al. (26), and Sangiovanni et al. (17) included only healthy participants. These studies extend coverage to several clinical groups, but many patient populations remain unrepresented. Evidence is still sparse or absent for common neurological and psychiatric conditions (e.g., neurologic-, affective-, and psychotic disorders), as well as for culturally diverse patients and those with motor or visual impairments, leaving substantial gaps for disorder-specific validation.

Over-reliance on automated models can affect clinical judgement and decision making. This has recently been exemplified in a recent multicenter study (36). The study found that endoscopists who routinely used AI for polyp detection became worse at standard (non-AI assisted) colonoscopy. These findings indicate that reliance on AI can affect clinical judgement and decision making. The question is not only whether to implement such technology, but how it is integrated into practice. This is highly relevant for successful implementation of automated scoring in neuropsychological assessment.

The core risk is over-reliance on the algorithmic output. Clinicians may bypass an independent visual review of drawings and accept the numerical score at face value, allowing the quantitative estimate to displace qualitative appraisal of copy strategy, organization, and error patterns. This risk is amplified when captured images are suboptimal, e.g. cropped, rotated, low contrast, or otherwise degraded (domain shift). These considerations argue for a human-in-the-loop workflow that treats the model’s output as an assistive second opinion rather than a replacement for clinical judgement. In short, the question is not only whether to implement such technology, but how it is integrated into practice.

## 5 Conclusion

Automated scoring has potential to enhance clinical neuropsychology, but safe adoption requires more than accuracy. Systems must be validated; their outputs should be interpretable and auditable; and deployment must include robust data-protection measures, bias monitoring, and clinician-controlled overrides. Ethical risks remain salient: unapproved secondary use (e.g., repurposing clinical drawings for training); “construct creep” can erode validity if models weight features outside the intended scoring construct; and black-box explanations may undermine trust, consent, and accountability. In this landscape, best practice is a human-in-the-loop workflow that treats automation as a supplementary option: accelerating routine scoring, flagging low-confidence or out-of-distribution cases, and preserving clinician judgment where it matters most. Current evidence supports an augment-not-replace role: automated scoring can shorten turnaround time, improve inter-rater consistency, and surface process information that would otherwise be lost, while clinicians integrate these outputs with history, examination, imaging, and patient priorities. Establishing guidelines for implementation is paramount.
