# Supplementary material for: Automated versus human scoring of the Rey-Osterrieth Complex Figure Test: a rapid review
Source: Front Psychiatry. 2026 Jan 14;16:1746720. doi: 10.3389/fpsyt.2025.1746720 (PMC12847431; doi:10.3389/fpsyt.2025.1746720)
Supplement: Supplementary file 1 [file Table1.docx]

Supplementary Material

# PRISMA flow chart

**Identification of studies via databases and registers**

Records removed *before screening*:

Duplicates removed (n = 61)

Records identified from:

Databases (n = 459)

- PubMed (n = 73)
- Web of Science (n = 386)

**Identification**

Records screened

(n = 398)

Records excluded**

(n = 377)

Reports sought for retrieval

(n = 21)

Reports not retrieved

(n = 0)

**Screening**

Reports excluded:

Did not meet inclusion criteria (n = 18)

Reports assessed for eligibility

(n = 21)

Additional eligible articles found (n = 2)

Bibliography search in eligible articles (n = 3)

Studies included in review

(n = 5)

**Included**

Source: Page MJ, et al. BMJ 2021;372:n71. doi: 10.1136/bmj.n71.

This work is licensed under CC BY 4.0. To view a copy of this license, visit <https://creativecommons.org/licenses/by/4.0/>
